# On the use of Parylene C polymer as substrate for peripheral nerve electrodes

**DOI:** 10.1038/s41598-018-24502-z

**Published:** 2018-04-13

**Authors:** Natàlia de la Oliva, Matthias Mueller, Thomas Stieglitz, Xavier Navarro, Jaume del Valle

**Affiliations:** 1 Institute of Neurosciences, Department of Cell Biology, Physiology and Immunology, Universitat Autònoma de Barcelona, and Centro de Investigación Biomédica en Red en Enfermedades Neurodegenerativas (CIBERNED), Bellaterra, Spain; 2 Laboratory for Biomedical Microtechnology, Department of Microsystems Engineering-IMTEK, Albert-Ludwig-University Freiburg, Freiburg, Germany; 3 Catalan Institute of Nanoscience and Nanotechnology (ICN2), CSIC and BIST, Campus UAB, Bellaterra, 08193 Barcelona Spain

## Abstract

Parylene C is a highly flexible polymer used in several biomedical implants. Since previous studies have reported valuable biocompatible and manufacturing characteristics for brain and intraneural implants, we tested its suitability as a substrate for peripheral nerve electrodes. We evaluated 1-year-aged *in vitro* samples, where no chemical differences were observed and only a slight deviation on Young’s modulus was found. The foreign body reaction (FBR) to longitudinal Parylene C devices implanted in the rat sciatic nerve for 8 months was characterized. After 2 weeks, a capsule was formed around the device, which continued increasing up to 16 and 32 weeks. Histological analyses revealed two cell types implicated in the FBR: macrophages, in contact with the device, and fibroblasts, localized in the outermost zone after 8 weeks. Molecular analysis of implanted nerves comparing Parylene C and polyimide devices revealed a peak of inflammatory cytokines after 1 day of implant, returning to low levels thereafter. Only an increase of CCL2 and CCL3 was found at chronic time-points for both materials. Although no molecular differences in the FBR to both polymers were found, the thick tissue capsule formed around Parylene C puts some concern on its use as a scaffold for intraneural electrodes.

## Introduction

Neural interfaces are the base of advanced neuroprostheses as they are responsible to bidirectionally communicate the nervous system with the mechanical prosthesis^1,2^. Neural interfaces should be able to record high-quality nerve signals to drive the prosthetic limb and to stimulate specific groups of axons within a nerve to evoke sensory feedback to control the prosthesis over long periods of time accurately. In order to achieve this communication, different types of electrodes for the use in peripheral nerves have been developed^3^, being the intraneural electrodes the most promising option to obtain a high selectivity of stimulation and recording with an acceptable invasiveness^4–6^.

However, after the implantation of any device in the body, an immune-mediated response – known as foreign body reaction (FBR) – occurs. The FBR is an immune-mediated response to any device implanted in the body. It is characterized by a first inflammatory phase followed by a tissue remodeling phase, which results in the encapsulation of the implant with immune cells and fibrotic tissue^7,8^ and it may finally lead to the failure of the device due to the lack of interaction with the tissue^9,10^.

Intraneural interfaces should have good biocompatibility and stability to remain inside the nerves for months or years, and the substrate material on which the electrode is made has an essential role in this maintenance^11^. It has to be biocompatible and biostable, avoiding the release of degrading particles that will induce an exacerbated inflammatory reaction. Another recognized problem is the mismatch of compliance between neural tissue and the implant material; indeed, reducing biomaterial stiffness can reduce the activation of immune cells leading to a less severe FBR^12^. Regarding the advances in biocompatible materials, several polymers have emerged as feasible options as substrates of flexible neural electrodes, such as polyimide and Parylene C, which also offer excellent insulation properties between adjacent metal tracks^13^.

Parylene C is a highly flexible polymer that has been used in many biomedical applications, e.g., as a coating for implants and electronic circuits^10,14^ as well as a substrate material for neural interfaces^15,16^, retinal implants^17^ and brain implants^13,18^ due to its good biocompatibility and biostability^18–21^. Moreover, unlike polyimide and other polymers used, Parylene C allows for thinner layers that could improve the implantation procedure and reduce the damage to the nerves^16,22^. Thus, Parylene C would be a good candidate to act as a scaffold of flexible interfaces for the peripheral nerve. However, the progression of the FBR to Parylene C implanted in the nerve has not been described yet and is mandatory to know the effect of Parylene C over long periods of time

In order to test whether Parylene C may be a suitable material for intraneural applications, test samples were aged, both *in vitro* and *in vivo*, and investigated chemically and mechanically. On the other hand, the cellular and molecular processes participating in the FBR to Parylene C mock devices implanted in the rat sciatic nerve have been characterized from 1 day up to 8 months. Finally, a comparison in the cytokine expression after implant has been performed with polyimide mock devices, as a standard polymer currently used in flexible neural interfaces^23–25^.

## Results

### Long-term stability of Parylene C substrates

**Figure 1:**
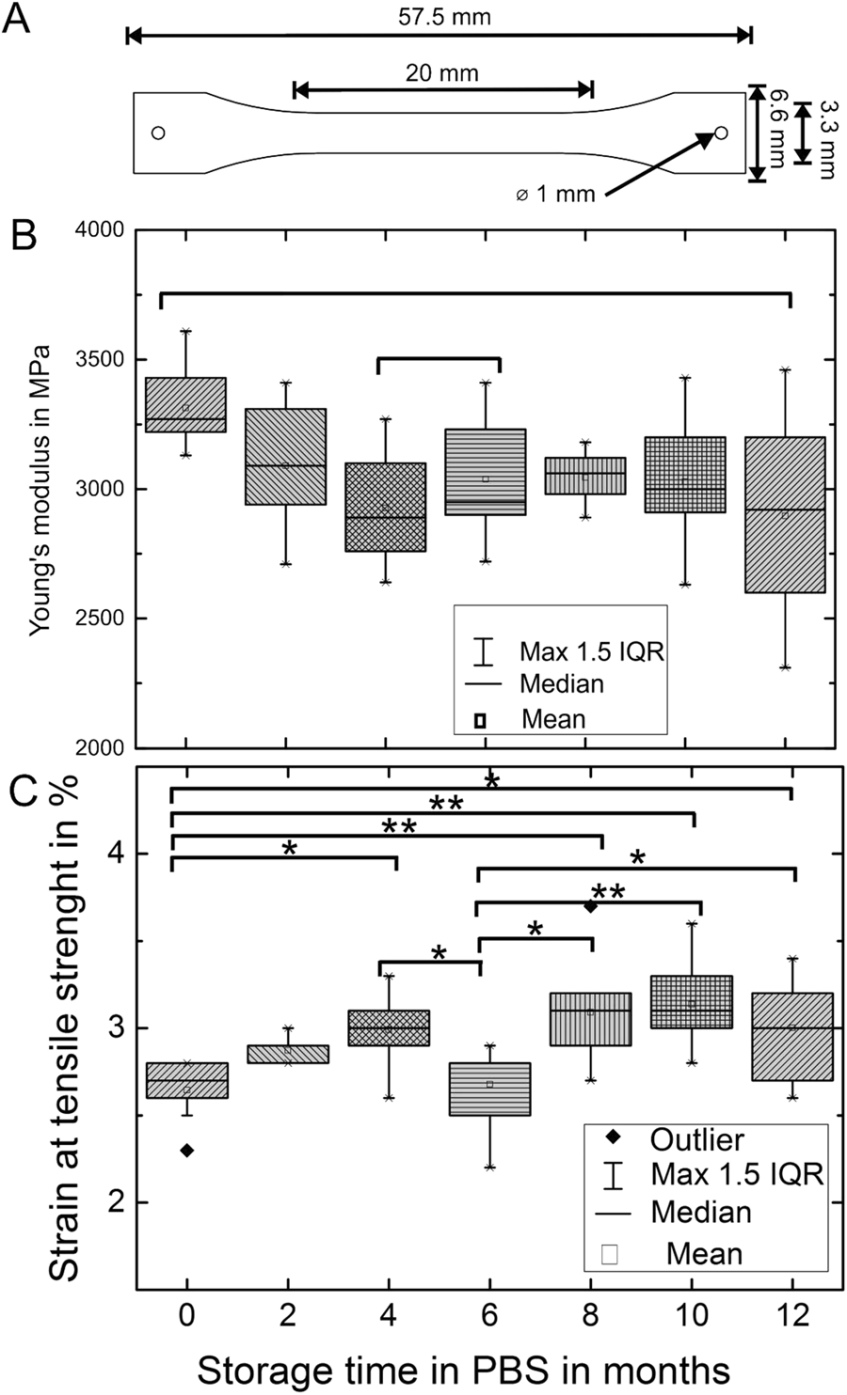
(**A**) Tensile testing samples, according to DIN EN ISO 527-3. (**B**) Young’s modulus of aged Parylene C samples as box plots. Depiction of 10 samples per measurement set. Maximum length of whiskers is 1.5 times the interquartile range (IQR). ANOVA: F(6,60) = 3.04, p-value = 0.01. Significance (Tukey): *p < 0.05. (**C**) Strain at tensile strength of aged Parylene C samples as box plots. Depiction of 10 samples per measurement set. Maximum length of whiskers is 1.5 times IQR ANOVA: F(6,60) = 7.39, p-value = 6.1*10^−6^. Significance (Tukey): *p < 0.05, **p < 0.001.

The mean Young’s modulus of an unaged sample was measured as 3.31 GPa and thus noticeable higher than the manufacturer’s value of 2.76 GPa. A general trend of decreasing Young’s modulus and rising divergence of the minimum and maximum values was found (Fig. 1B). However, an exception was observed at month 8, thus the correlation needs to be regarded as an estimation. ANOVA testing revealed a statistically significant difference between the grouped samples.

The mean initial strain at tensile strength was measured as 2.64%, in good accordance with the manufacturer’s data. Mean initial strain at break was measured as 8.63%, which is way below the expected value. Over time a slight increase in the strain was observed (Fig. 1C). All mechanical properties are presented in Table 1.

**Table 1:** Mechanical properties of aged Parylene C.

	0 mon.	2 mon.	4 mon.	6 mon.	8 mon.	10 mon.	12 mon.
Young’s modulus *E* in GPa	3.31	3.09	2.93	3.04	3.04	3.03	2.90
Max	3.61	3.41	3.27	3.41	3.18	3.43	3.46
Min	3.13	2.71	2.64	2.72	2.89	2.63	2.31
Elongation at tensile str. *ε*_*y*_ in %	2.6	2.9	3.0	2.7	3.1	3.1	3.0
Max	2.8	3.0	3.3	2.9	3.7	3.6	3.4
Min	2.3	2.8	2.6	2.2	2.7	2.8	2.6

Samples were stored in PBS up to 12 months (mon.). Evaluation over n = 10 samples.

**Figure 2:**
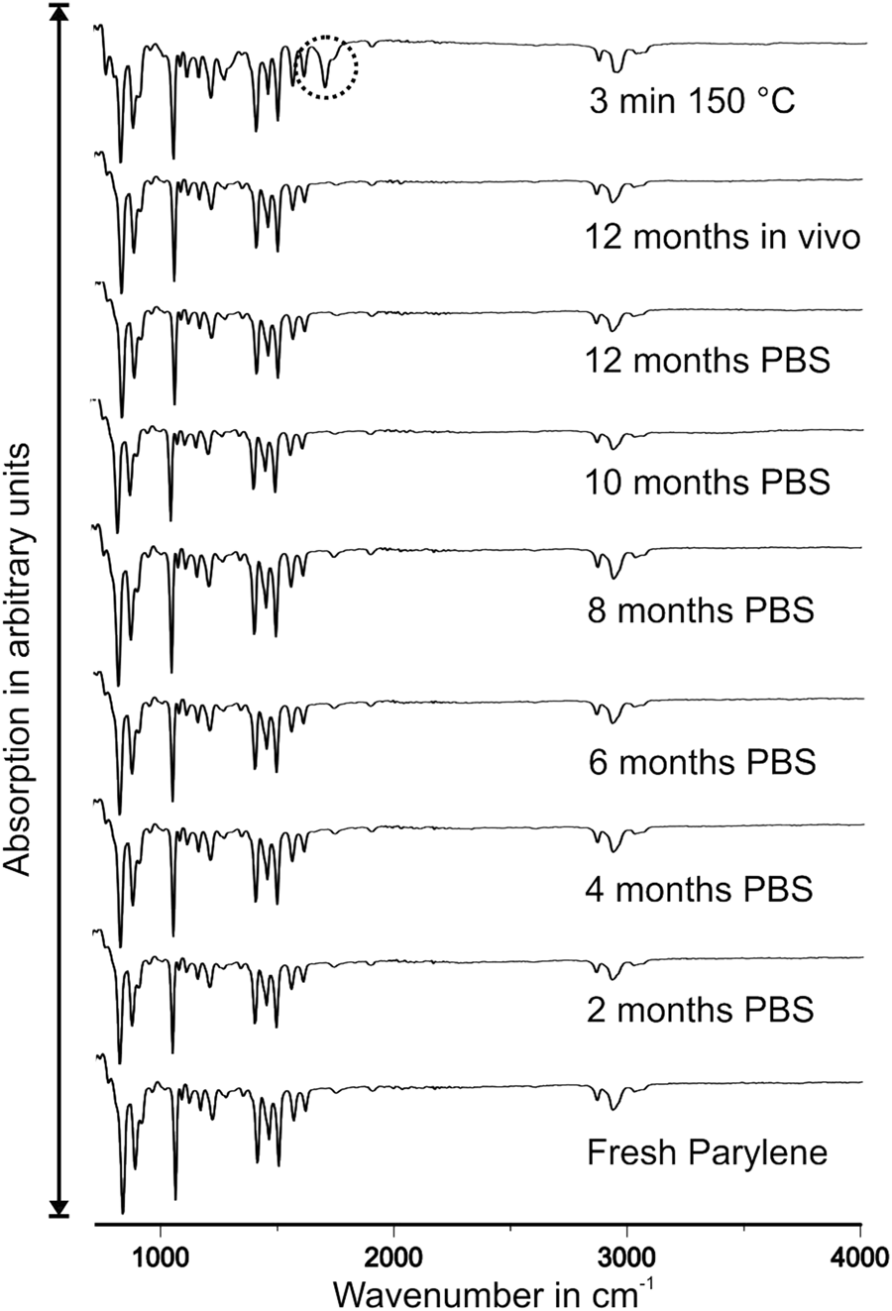
FTIR spectra of fresh, aged, explanted and intentionally oxidized Parylene C samples. The absorption is presented in arbitrary units. Only oxidized Parylene C exhibits an additional peak.

The spectra of fresh, aged, explanted and intentionally oxidized Parylene C are depicted in Fig. 2. All peaks were in good accordance with the characteristic FT-IR peaks of Parylene C^26^. Parylene C has very little peaks outside the fingerprint region (500 cm-1 to 1450 cm-1). Whereas the intentionally, at low temperature, oxidized sample exhibited an expected oxidation peak, all other samples did not possess any new peaks, correlating to the change or addition of functional groups or degradation.

### Nerve response and capsule formation

Electrophysiological tests showed no differences in gastrocnemius CMAP amplitude and latency between implanted and sham animals. Moreover, the results of the Von Frey test showed no changes in pain thresholds, and the SFI values remained within normal locomotor values for the implanted and the sham groups (Supplementary Fig. 1A–D).

**Figure 3:**
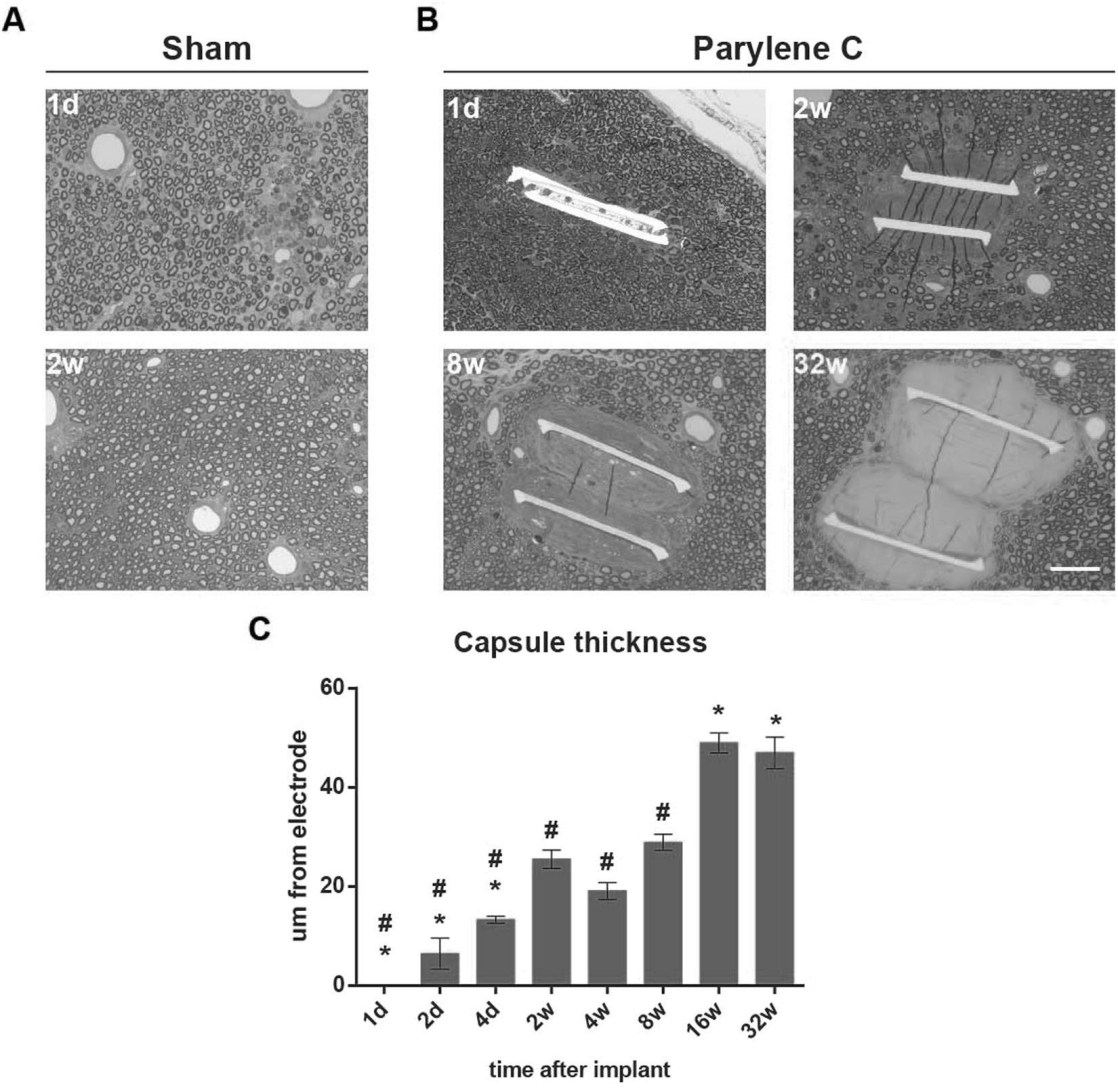
Progression of tissue capsule formation around the Parylene C device implanted in the nerve. Representative light microscopy images of (**A**) sham nerves after 1 day and 2 weeks in which no tissue deposition can be appreciated, (**B**) Implanted nerves with progressive tissue capsule around the device at 1 day, 2, 8 and 32 weeks. (**C**) Increase in tissue capsule thickness along time. Scale bar = 50 µm. *p < 0.05 vs 2w. ^#^p < 0.05 vs 16w.

Light microscopy observations showed no alterations in the fascicular architecture compared to control nerves, and the total number of myelinated fibres did not show significant differences in both implanted and sham nerves at the different time-points analysed, from 1 day to 32 weeks (Supplementary Fig. 1E–G). Detailed evaluation of the tissue deposition around the implanted devices was performed under light and transmission electron microscopy. In sham-operated animals, no scar tissue could be identified at any time point (Fig. 3A). In contrast, clear tissue deposition was observed around the Parylene C device implant (Fig. 3B). The thickness of the tissue capsule increased up to 30 µm during the first two weeks of implant, followed by stabilization and possible compaction at 4 weeks. However, the tissue deposition showed a later increase from 8 to 16 weeks, when it achieved a maximum of 50 µm thickness, and then appeared stabilized at 32 weeks post implant (Fig. 3C).

**Figure 4:**
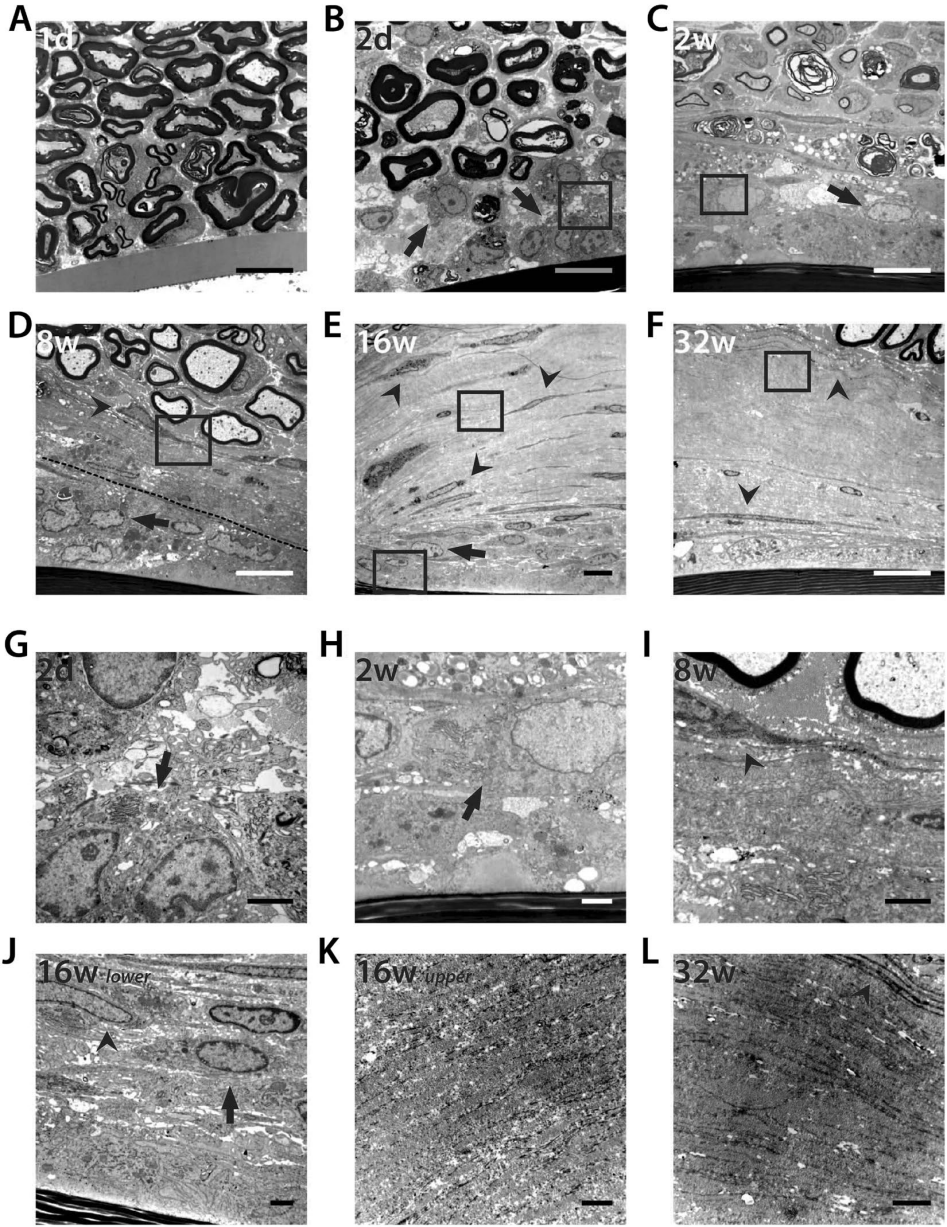
Detailed evaluation of tissue capsule by TEM. (**A**–**F**) Representative images of the tissue capsule at 1 and 2 days, 2, 8, 16 and 32 weeks postimplant. Scale bar = 10 µm. At (A) 1 day, (B) 2 days and (**C**) 2 weeks only amoeboid cells (arrows) were seen in the vicinity of the device. From (**D**) week 8 two different zones can be distinguished in the capsule (dotted line), with spindle-shaped cells localized at the periphery of the capsule (arrowheads). After (**E**) 16 and (**F**) 32 weeks post-implant, the capsule was mainly formed by spindle-shaped cells and collagen fibers (insets, G–L). Insets show the progressive change from (**G**–**H**) amoeboid cells at early time points to (**I**–**L**) spindle-shaped cells, together with an increase in (**K**–**L**) collagen deposition from 8 weeks. Scale bar = 2 µm.

Early changes in the vicinity of the implants were observed by TEM during the first days following the implantation. After one day, axons were still in close contact with the device and no foreign cells were seen near the device (Fig. 4A). However, by day 2, amoeboid cells appeared in the space between axons and the device embedded in a loose and disorganized matrix (Fig. 4B). At 2 weeks, the capsule surrounding the Parylene C implant appeared full of more organized amoeboid cells (Fig. 4C). There were some figures of axonal degeneration near the implant and macrophagic cells engulfing the myelin debris. After 8 weeks, spindle-shaped cells started to appear at the edge of the capsule surrounded by collagen fibrils (Fig. 4D,I). At this time, two zones could be differentiated in the capsule: an inner area with compacted amoeboid cells in contact with the device, and an outer area where spindle-shape cells were near the axons. This pattern remained with time; by 16 weeks post-implant, the inner area remained similar, and the outer zone appeared thicker and with more spindle-shaped cells embedded in a dense collagen matrix (Fig. 4E,J,K). After 8 months post-implant, the tissue around the device appeared as a compact and organized matrix with spindle-shaped cells and some FBGCs in the inner zone (Fig. 4F,L).

### Cellular characterization of the FBR

**Figure 5:**
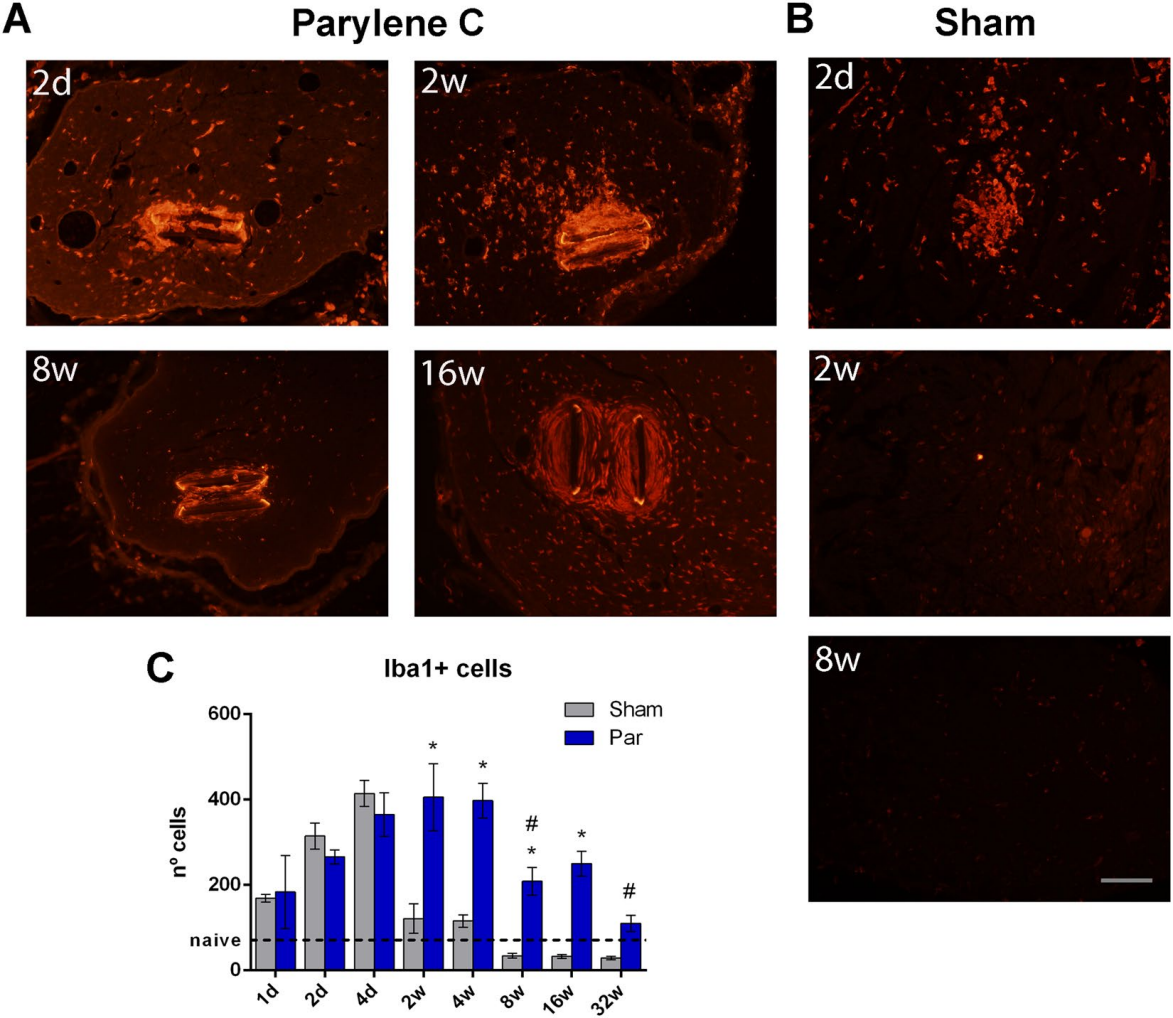
Iba1 labeling of infiltrating macrophages in the tibial nerve. Representative images of (**A**) implanted and (**B**) sham nerves at different time-points. Scale bar = 100 µm. (**C**) Number of iba1 positive cells in the whole tibial nerve along time. *p < 0.05 vs sham animals. ^#^p < 0.05 vs 2w implanted.

Macrophage infiltration in the nerve was analyzed by Iba1 labeling as a measure of the inflammatory response. In the sham implanted tibial nerves, the number of Iba1+ cells increased during the first days after implant but resolved to near normal levels at 2 weeks. In contrast, in Parylene C implanted nerves the number of iba1+ cells persisted at a high level from 4 days to 4 weeks after implant (Fig. 5). Besides, resolution of the inflammatory infiltration started at 8 weeks, and it was not complete even at 32 weeks. Thus, there was an early inflammatory reaction from the surgery, but the device implanted caused a persistent infiltration of macrophages and inflammatory cells in the nerve with delayed resolution.

**Figure 6:**
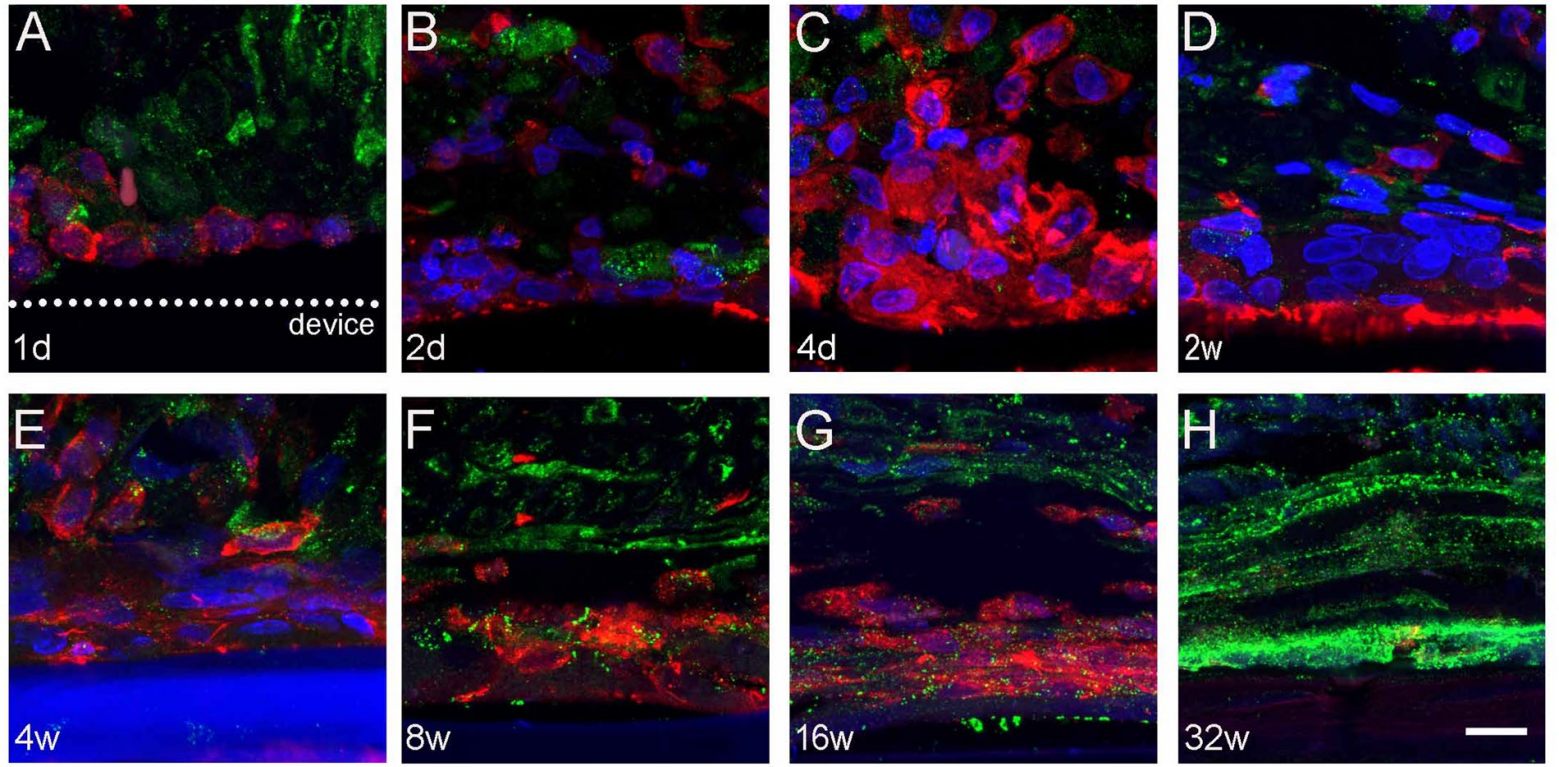
Cellular composition of the capsule. Representative confocal microscopy images labeling against Iba1 and CD90 markers. Only iba1+ macrophages were observed from (**A**) 1 day to (**E**) 4 weeks post-implant. From (**E**) 8 to (F) 16 weeks, CD90+ fibroblasts were located in the periphery of the capsule and by (**G**) week 32w, CD90+ fibroblasts occupied most of the area of the capsule. Iba1 in red, CD90 in green, DAPI in blue. Scale bar = 5 µm.

To determine the cellular mediators of the FBR to Parylene C devices, we used immunolabeling of the capsule tissue around the implant. Iba1 positive cells rapidly surrounded and started to form the capsule around the Parylene C device (Fig. 6). From 4 days to 4 weeks post-implant only Iba1 positive amoeboid cells were found around and in direct contact with the device. From 8 to 16 weeks post-implant, iba1+ cells became more organized and flattened. On the other hand, CD90+ fibroblast cells started to appear at the edge of the capsule at 8 weeks. By week 32 post-implant, only CD90 positive cells were observed in the capsule around the implant, without presence of Iba1+ cells (Fig. 6).

The presence of FBGC in the capsule was also analyzed as a hallmark of the FBR. These cells are the result of macrophage fusion when those are unable to phagocytize large devices and adopt an extracellular degradation strategy^27^. FBGC appeared soon after the implantation, and its number increased until 2 weeks, when it slightly decreased and remained stable (Supplementary Fig. 2). However, there were no significant variations in its size at any studied time point.

### Molecular environment of the FBR

To further characterize the immune response to devices implanted in the peripheral nerve, cytokines and TGFβ levels were quantified using Luminex technology from 6 hours to 8 weeks post-implantation. This analysis was also made for polyimide devices for comparison.

**Figure 7:**
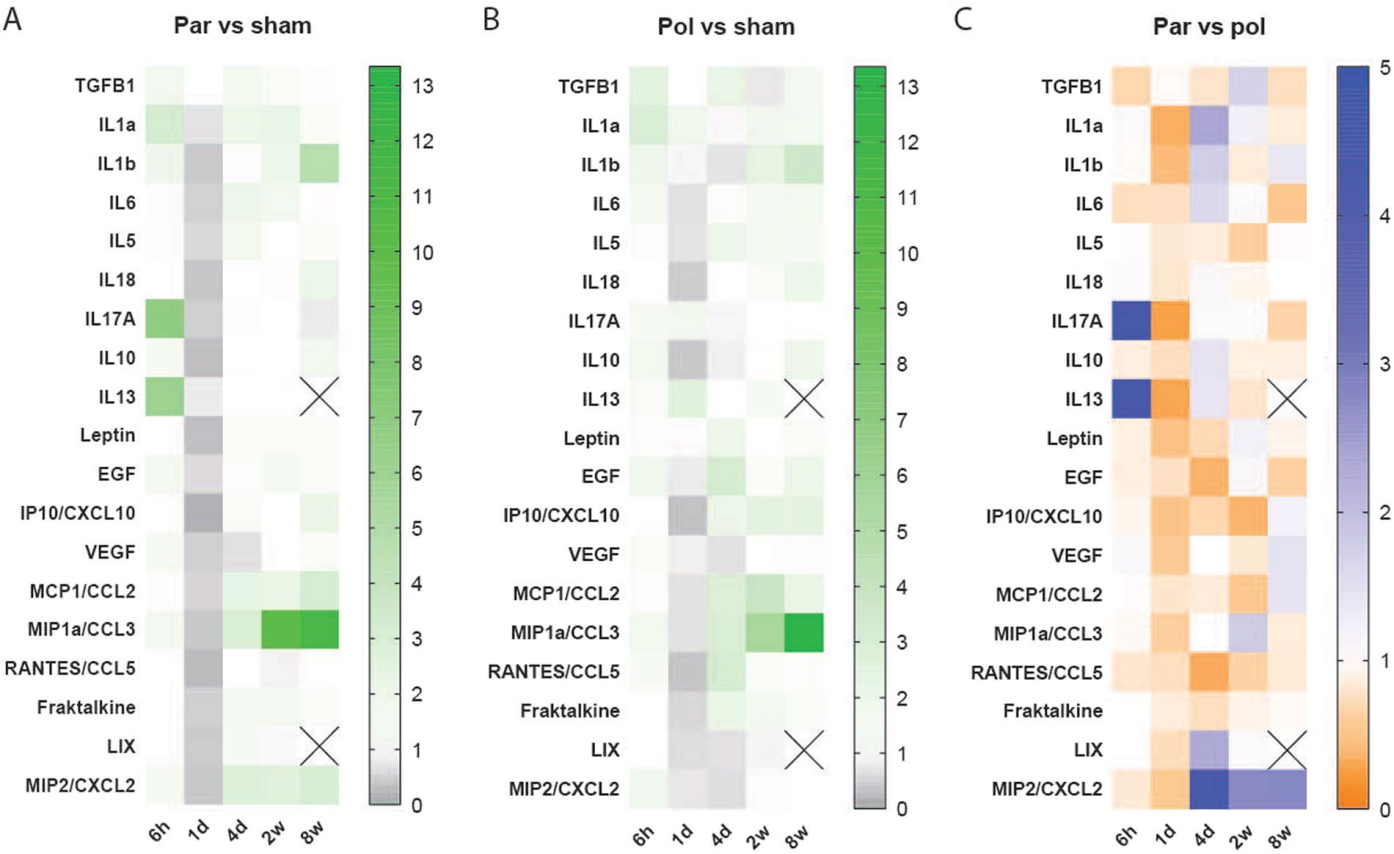
Cytokines levels obtained by multiplex analysis. Heatmaps showing the changes in several proteins at different time points in (**A**) Parylene C and (**B**) polyimide implanted nerves versus sham nerves. Results are expressed as the mean of the ratio between each group and sham values. (**C**) Heatmap representing the changes in Parylene C implanted nerves with respect to polyimide implanted nerves. Results expressed as the mean of the ratio between Parylene C and polyimide values. Crossed out squares mean no protein detected.

On the one hand, the analysis showed an increase for most of the studied factors at 6 hours and 1 day after surgery in the implanted and sham nerves in comparison to intact nerves, which was recovered to control values from 4 days onwards (Supplementary Fig. S3). On the other hand, there were no differences in the levels of the main pro-inflammatory, anti-inflammatory or tissue-remodelling factors between Parylene C or polyimide implanted nerves and the sham animals (Fig. 7 and Supplementary Fig. S4). However, an increase in the levels of CCL2 and CCL3 was observed for both materials in comparison to sham animals at chronic time points. Moreover, there were not any different factor at any time point between the responses to Parylene C or polyimide devices (Fig. 7 and Supplementary Fig. S4C). Some interleukins, such as IL1a, IL1b, IL17A, and IL13, showed very high values at 6 hours and 1 day after implant. At chronic time points, there were no apparent differences between materials in any of the studied cytokines. MIP2/CXCL2 was the only one to show different levels between Parylene C and polyimide from 4 days to 8 weeks, although they were no statistically significant.

## Discussion

After the implantation of any device in the body, several cellular and molecular changes such as protein adsorption, cellular infiltration, tissue deposition and encapsulation of the implanted device occur^7,8^. In the case of neural interfaces, they need direct interaction with neural fibers to stimulate or record from them, and the encapsulation due to the FBR induces a gradual decline of neural electrodes function^16,18^. Novel strategies such as the investigation of different polymers and coatings^28–30^ have been developed to overcome this problem. In this regard, Parylene C has been used as an insulator^13,31^ and as a substrate^16,32–34^ in neural applications^35^. Here we have focused on the characterization of the FBR to Parylene C devices to determine its suitability for peripheral nerve interfaces.

The mechanical parameters of Parylene C deviated from the manufacturer’s data. Whereas Young’s modulus was higher, the strain at tensile strength was in good accordance with the expected value. A possible explanation for the deviation might be in the low deposition pressure of 20 mTorr used in sample fabrication, in contrast to the recommended pressure of 60 mTorr by the supplier. As discussed by Hassler *et al*. the general conformity of Parylene C is dependent from the actual deposition parameters^31^. Concerning the elongation, Song *et al*. could prove a strong dependency from deposition pressures between 25 and 45 mTorr^36^.

Ageing and *in vivo* experiments had no apparent chemical effect on Parylene C, which would have been observable by an oxidation peak or missing bands in the FTIR spectra. However, an influence on the mechanical properties was observed for aged samples. Young’s modulus slightly decreased over time, whereas the strain at tensile strength slightly increased. For a repetition of the *in vivo* studies, another standard with smaller tensile testing samples would be recommended.

Regarding the functional properties of the nerves with Parylene C implanted devices, there were no alterations in the nerve conduction, nociception, and locomotion. Moreover, no changes in the nerve architecture and the total number of myelinated axons were found due to surgery or the device implanted. Taking into account that the model used is a longitudinal device, these results are in agreement with previous studies with thin film longitudinal intrafascicular electrodes^25^. Besides, these results confirm the biocompatible characteristics of Parylene C as there were no harmful effects on nerve function^19,37^.

Several studies, mainly using subcutaneous and intraperitoneal implants^7,8,27^ have shown that the FBR has a first inflammatory phase followed by a tissue remodelling phase, and a similar pattern has been observed for the FBR in peripheral nerve implant models^38–41^. First, macrophages rapidly infiltrate the nerve after the implantation and start to surround the device after 1 day. These infiltrating cells generate a cellular capsule that enlarges until 2 weeks, coinciding with the peak of the inflammatory reaction. In parylene C implanted nerves, nerve inflammation is resolved by 8 weeks, but the thickness of the capsule increases up to 16 weeks with a change in its cellular composition and fibroblasts appearing in the edge of the tissue capsule by week 8. However, although the response to intraneural implants of Parylene C is similar to that recently described for polyimide implants with the same physical shape and dimensions^39^, a thicker tissue deposition has been found. Moreover, macrophages remained for a longer time in the inner zone of the capsule and more fibroblasts appeared around Parylene C implants in comparison with polyimide devices. Thus, the specificities and time evolution of the FBR should be studied for each material used and the tissue in which the device is implanted^11^.

In light of these results, it seems that Parylene C is stimulating a different pattern of FBR than polyimide devices. The general pattern of the FBR for non-degradable implants is a switch from a pro-inflammatory to an anti-inflammatory and tissue deposition environment. A direct relationship seems to occur between these two phases^42,43^. However, in the case of Parylene C devices, while the inflammatory reaction decreased, evidenced by the disappearance of Iba1 positive cells in the whole nerve after 8 weeks of the implant, the tissue deposition continued increasing, and macrophages remained in the tissue capsule to the implant.

Molecular analyses were performed to clarify these particular differences in the FBR between the two materials. However, no differences in the expression of several inflammation-related cytokines were observed between Parylene C and polyimide responses at any time point. This could be explained by the fact that highest levels of cytokines are generally found within the first hours or days after injury^44–46^. In our model, the injury due to the implantation is resolved by about 2 weeks, and by then, the levels of cytokines are already decreased. By week 8, inflammation of the nerve is almost resolved, cytokines levels have decreased, and the contribution of each material to cytokines production is not detected in the whole homogenized nerve. Hence, further analyses are needed to evaluate the macrophage activation in the local area surrounding the Parylene C implants. Even though, our results point at CCL3 and CCL5 as potential targets to reduce the tissue deposition around implanted devices. It has been shown that CCL3/MIP1a and CCL5/RANTES recruit polymorphonuclear cells and macrophages^44,45,47^, thus increasing the inflammatory phase of the FBR. On the other hand, CXCL2 levels are also increased in Parylene C in comparison to polyimide implanted nerves at chronic time points, although no significantly.

Differences in FBR can have their origin in the different chemical structure of polyimide and Parylene C. While the aromatic rings in the polyimide are in stable configurations, Parylene C is known to have non-saturated radicals at the carbon atoms that might result in different surface potentials (Zeta-potential) that influence interaction with proteins at the material-tissue interface^48,49^. Differences in polymer surface would also explain these results. Jones and co-workers showed that hydrophilic surface promotes less cellular adhesion but a higher cytokine production^50^. Although both materials show a hydrophobic behaviour with contact angles ranging from 80 to 100°, Parylene C can be considered more hydrophilic than polyimide^19,51^, which could explain the higher matrix deposition due to a higher inflammatory environment.

In summary, the implantation of a Parylene C device mimicking a longitudinal nerve electrode does not cause noticeable damage to the peripheral nerve, and shows good biocompatibility. However, the generated FBR is stronger against Parylene C than to polyimide-based devices, that results in a thicker tissue capsule around the implanted device. Even its sound biocompatible and functional characteristics showed in this and other studies^15,16,18,19^, the increased tissue deposition in comparison to polyimide would rule Parylene C out as a neural substrate. Moreover, our results point out the need for specific studies for each material and each implanted tissue, regarding the differences that can arise after chronic implants.

## Material and Methods

### Mechanical and chemical investigation *in vitro*

Mechanical and chemical evaluation of aged Parylene C was performed with 10 µm thick samples according to DIN EN ISO 527-3 “Plastics - Determination of tensile properties - Part 3: Test conditions for films and sheets” (DIN EN ISO 527-3:2003-07 2003) (Fig. 1A). From the different possible designs, type 1B was scaled down by a factor of three, which enables tensile testing as well as subcutaneous *in vivo* implantation.

The samples were stored in PBS at 37 °C for up to 12 months in a dark environment. From each set 10 samples were taken out of the PBS and allowed to dry overnight. Each sample was fitted with a small piece of Kapton tape at the ends and clamped between the clamp jaws of a tensile tester (Z2.5, Zwicki-Line, Zwick/Roell, Ulm, Germany). The clamp jaws were additionally equipped with abrasive paper to provide a stronger grip on the thin samples. Force measurements were detected with a 50 N load cell (KAP-S, A.S.T. GmbH, Wolznach, Germany). After applying an initial load of 2 MPa, the clamps moved apart with a velocity of 5 mm/min. Even though it is common practice to measure Young’s modulus by fitting a straight line between the stress at 0.05% and 0.25% of strain, it was measured between 0.25% and 0.5% due to the low initial load^52^. Furthermore, the strain on tensile strength was derived from the measured stress-strain diagrams.

The aspect of possible chemical degradation was investigated by Fourier-transform infrared spectroscopy (FTIR). An FTIR (2000 FT-IR Scimitar Series, Varian Inc., Palo Alto, California, United States) was fitted with an attenuated total reflectance (ATR) unit (MKII Golden Gate, Specac Ltd., Orpington, Kent, United Kingdom) and flushed with nitrogen. Measurements were performed against a nitrogen atmosphere, constantly flushing for 15 minutes. Additionally, fresh Parylene C samples were heated to 150 °C for three minutes under normal atmosphere, to act as a slightly degraded sample.

### Animals and surgical procedures

Female Sprague-Dawley rats (250–300 g, n = 6–8/group) were anaesthetised using ketamine-xylazine (90/10 mg/kg i.p.). The sciatic nerve was surgically exposed at the midthigh and Parylene C or polyimide double devices (20 mm long, 200 µm wide and 10 µm thick), similar to tfLIFE electrodes, were longitudinally implanted in the tibial branch as before^25,53^. A group of sham animals underwent the same procedure but leaving no device inside the nerve.

Animals were housed at 22 ± 2 °C under a 12:12 h light cycle with food and water ad libitum. All animal experiments were approved by the Ethical Committee of the Universitat Autònoma de Barcelona in accordance with the European Communities Council Directive 2010/63/EU.

At 6 hours, 1, 2, 4 days, 2, 4, 8, 16, and 32 weeks post-implant, animals were deeply anesthetized with an overdose of pentobarbital and transcardially perfused with 4% paraformaldehyde in PBS or saline. The sciatic nerve including the device was harvested for histological or molecular analysis.

Additionally, a set of 10-µm-thick samples according to DIN EN ISO 527–3 were implanted subcutaneously in the back of another group of rats to evaluate the chemical and mechanical properties of aged Parylene C after 1 year of implant. Animals dorsum was shaved and disinfected with 70% alcohol and povidone iodine. Afterwards, 3 incisions were done on the back, with a distance of 2 cm between incisions and the skin surrounding the incisions was separated from the dorsal subcutaneous tissue in order to prepare three implant pockets per animal. The pieces were placed and fixed inside each pocket.

The polyimide bulk material was fabricated from the liquid precursor U-varnish-S by UBE Industries (Tokio, Japan). In the liquid state, it can be spin-coated and, after evaporation of the solvent, cured at 350 °C^54^. The Parylene C bulk material was deposited with the Gorham process from the dimer di-para-xylylene C within a parylene coater (both from SCS Speciality Coating Systems Inc., USA)^16^.

### Functional evaluation

To evaluate functional properties of the implanted nerves, nerve conduction, algesimetry and locomotion tests were performed at different time points after implant. Nerve conduction test was performed by stimulating the sciatic nerve proximally with single electrical pulses and recording the compound muscle action potentials (CMAPs) of gastrocnemius medialis (GM) muscle as previously described^4,25^. Pain threshold to mechanical stimuli was assessed by means of an electronic Von Frey algesimeter (Bioseb, Chaville, France). The hindpaw plantar surface was stimulated by applying a non-noxious pointed probe, slowly increasing the pressure until the rat withdrew the paw in response to the stimulus^55^. The walking track test was performed to assess locomotor function after the implant. The plantar surface of the hind paws was painted with black ink, and the rat was left to walk along a corridor freely. The print length, the distance between the 1st and the 5th toes and between the 2nd and the 4th toes were measured to calculate the Sciatic Functional Index (SFI)^56^.

### Morphological evaluation

To evaluate the microstructure of implanted nerves and the myelinated axons, a segment of the tibial nerve was sectioned in semithin sections (0.5 µm-thick) and stained with toluidine blue after epon resin embedding. To estimate the number of myelinated fibers in the tibial nerve, sections were examined by light microscopy and axons were counted in images at 100×, chosen by systematic selection and representing at least the 30% of the nerve cross-section area. The whole area of the tibial nerve was measured in 4× images. Using the same sections, the thickness of the tissue capsule around the devices implanted was measured as the distance between each side of the device and the closest myelinated axon using ImageJ software.

Transmission electron microscopy (TEM) was performed to analyse the tissue and the collagen deposition around Parylene C implanted devices. Ultrathin sections (70 nm) were cut, mounted on formvar 200 mesh copper grids and contrasted with uranyl acetate/lead citrate. Images of the area with the device were taken using a TEM microscope (JEM 1400) to evaluate the encapsulating tissue at different time points.

### Immunohistochemistry

Another nerve segment containing the device implanted was serially cut (15 µm) with a cryostat. Slides were incubated overnight at 4 °C with primary antibodies anti-iba1 (Wako, 1:500) for macrophages and anti-CD90 (BD Pharmingen, 1:150) for fibroblasts. Slides were then washed and incubated with secondary antibodies for 1 h at room temperature, and mounted with mowiol containing DAPI (0,1 μg/ml).

To quantify the number of infiltrating Iba1+ macrophages, images of the whole tibial nerve were taken with an epifluorescence microscope BX51 (Olympus) attached to a DP73 digital camera (Olympus) and equally treated to adjust brightness and contrast. The background was subtracted and a threshold of detection and binarization was applied using ImageJ software. Iba1 positive cells in the whole tibial nerve, excluding the implanted device and tissue surrounding, were counted using the plugin “Analyze Particles” of ImageJ^39^.

### Haematoxylin-eosin staining

To determine the amount of foreign body giant cells (FBGCs) around the implant, cryostat sections were immersed in hematoxylin Harris solution for 7 min and washed in water followed by 1% HCl in ethanol solution for 20 sec. Sections were washed again with water and stained with Eosin Y for 5 min, dehydrated with series of graded ethanol rinses and mounted with DPX. The number of FBGC was counted under the microscope in each stained section and expressed as FBGC per mm of implant width. Moreover, pictures of every FBGC were taken, and the diameter was measured with ImageJ.

### Cytokine protein expression

Other animals were transcardially perfused with sterile saline, and 1 centimetre of the sciatic nerve including the implanted device was taken after 6 hours, 1 and 4 days, 2 and 8 weeks post-implant, snap-frozen with liquid nitrogen and kept at −80 °C. Sciatic nerves were homogenized with HEPES buffer and the protein concentration was determined using Pierce BCA assay kit. To ensure equal amounts of protein, all samples were diluted with HEPES to 4 µg/µl. Protein levels of a panel of cytokines (EGF, CCL11, Fraktalkine/CX3CL1, GCSF, GMCSF, CXCL1, IFNγ, IL1α, IL1β, IL2, IL4, IL5, IL6, IL10, IL12, IL13, IL17A, IL18, IP10/CXCL10, Leptin, LIX/CXCL6, MCP1/CCL2, MIP1α/CCL3, MIP2/CXCL2, RANTES/CCL5, TNFα, VEGF-A) and TGFβ (TGFβ1, TGFβ2, TGFβ3) were analyzed using the Milliplex map Rat Cytokine/Chemokine magnetic bead panel (RECYMAG65PMX27BK, Merck Millipore) and the Milliplex map TGFβ magnetic bead 3 Plex Kit (TGFBMAG-64K-03, Merck Millipore). Proteins with levels lower than detection limit are not shown.

### Statistical analysis

Reported values show the mean ± SEM. Differences between groups or times were analyzed by one or two-way ANOVA followed by Bonferroni post hoc tests (GraphPad Prism) for the animal experiments. Post-hoc Tukey’s range test was conducted for the mechanical experiments if the ANOVA showed statistical significance to rule out which groups were statistically different. Statistical significance was considered at p < 0.05. For protein levels, results are expressed as the ratio with intact, sham or polyimide values in heatmaps.

### Data availability

All data generated and analyzed during the current study are available from the corresponding author on reasonable request.

## Electronic supplementary material

Supplementary Figures

## References

[1] Navarro X (2005). A critical review of interfaces with the peripheral nervous system for the control of neuroprostheses and hybrid bionic systems. J. Peripher. Nerv. Syst..

[2] Tyler, D. J., Polasek, K. H. & Schiefer, M. A. *Peripheral Nerve Interfaces*. *Nerves and Nerve Injuries* **2** (Elsevier Ltd., 2015).

[3] del Valle J, Navarro X (2013). Interfaces with the peripheral nerve for the control of neuroprostheses. Int. Rev. Neurobiol..

[4] Badia J (2011). Comparative analysis of transverse intrafascicular multichannel, longitudinal intrafascicular and multipolar cuff electrodes for the selective stimulation of nerve fascicles. J. Neural Eng..

[5] Oddo CM (2016). Intraneural stimulation elicits discrimination of textural features by artificial fingertip in intact and amputee humans. Elife.

[6] Raspopovic S (2014). Restoring natural sensory feedback in real-time bidirectional hand prostheses. Sci. Transl. Med..

[7] Anderson JM, Rodriguez A, Chang DT (2008). Foreign body reaction to biomaterials. Seminars in Immunology.

[8] Ward KW (2008). A review of the foreign-body response to subcutaneously-implanted devices: the role of macrophages and cytokines in biofouling and fibrosis. J. diabetes Sci. Technol..

[9] Yu B, Ju Y, West L, Moussy Y, Moussy F (2007). An Investigation of Long-Term Performance of Minimally Invasive Glucose Biosensors. Diabetes Technol. Ther..

[10] Chen, X. & Young, D. Robust implantable blood pressure sensor packaging for long-term laboratory animals monitoring. in *IEEE SENSORS* 1–3 (2016).

[11] Boddupalli A, Zhu L, Bratlie KM (2016). Methods for Implant Acceptance and Wound Healing: Material Selection and Implant Location Modulate Macrophage and Fibroblast Phenotypes. Adv. Healthc. Mater..

[12] Blakney AK, Swartzlander MD, Bryant SJ (2012). The effects of substrate stiffness on the *in vitro* activation of macrophages and *in vivo* host response to poly(ethylene glycol)-based hydrogels. J. Biomed. Mater. Res. A.

[13] Ordonez J, Schuettler M, Boehler C, Boretius T, Stieglitz T (2012). Thin films and microelectrode arrays for neuroprosthetics. MRS Bull..

[14] Haggren T (2017). Nanowire encapsulation with polymer for electrical isolation and enhanced optical properties. Nano Res..

[15] Christensen, M. B. & Tresco, P. A. The foreign body response and morphometric changes associated with mesh-style peripheral nerve cuffs. *Acta Biomater*. 14–21 (2017). 10.1016/j.actbio.2017.11.059 29223703

[16] Mueller M (2017). Rapid prototyping of flexible intrafascicular electrode arrays by picosecond laser structuring. J. Neural Eng..

[17] Yue L, Weiland JD, Roska B, Humayun MS (2016). Retinal stimulation strategies to restore vision: Fundamentals and systems. Progress in Retinal and Eye Research.

[18] Lecomte A, Degache A, Descamps E, Dahan L, Bergaud C (2017). *In vitro* and *in vivo* biostability assessment of chronically-implanted Parylene C neural sensors. Sensors Actuators B Chem..

[19] Chang TY (2007). Cell and Protein Compatibility of Parylene-C Surfaces. Langmuir.

[20] Kim BJ, Meng E (2016). Micromachining of Parylene C for bioMEMS. Polym. Adv. Technol..

[21] Leung BK, Biran R, Underwood CJ, Tresco PA (2008). Characterization of microglial attachment and cytokine release on biomaterials of differing surface chemistry. Biomaterials.

[22] Mueller, M., Boehler, C., Jaeger, J., Asplund, M. & Stieglitz, T. A double-sided fabrication process for intrafascicular parylene C based electrode arrays. In *38th Annual International Conference of the IEEE Engineering in Medicine and Biology Society (EMBC)* 2798–2801 (2016). 10.1109/EMBC.2016.7591311 28268899

[23] Rubehn B, Stieglitz T (2010). *In vitro* evaluation of the long-term stability of polyimide as a material for neural implants. Biomaterials.

[24] Cutrone A (2015). A three-dimensional self-opening intraneural peripheral interface (SELINE). J. Neural Eng..

[25] Lago N, Yoshida K, Koch KP, Navarro X (2007). Assessment of biocompatibility of chronically implanted polyimide and platinum intrafascicular electrodes. IEEE Trans. Biomed. Eng..

[26] Nowlin TE, Smith DF, Cieloszyk GS (1980). Thermal oxidative stability of poly-p-xylylenes. J. Polym. Sci. Polym. Chem. Ed..

[27] Sheikh Z, Brooks PJ, Barzilay O, Fine N, Glogauer M (2015). Macrophages, foreign body giant cells and their response to implantable biomaterials. Materials.

[28] Sommakia S, Lee HC, Gaire J, Otto KJ (2014). Materials approaches for modulating neural tissue responses to implanted microelectrodes through mechanical and biochemical means. Curr. Opin. Solid State Mater. Sci..

[29] Yu, T., Tutwiler, V. J. & Spiller, K. The Role of Macrophages in the Foreign Body Response to Implanted Biomaterials. In *Biomaterials in Regenerative Medicine and the Immune* System (ed. Santambrogio, L.) 17–34 (Springer International Publishing, 2015).

[30] Sadtler K (2016). Design, clinical translation and immunological response of biomaterials in regenerative medicine. Nat. Rev. Mater..

[31] Hassler C, Von Metzen RP, Ruther P, Stieglitz T (2010). Characterization of parylene C as an encapsulation material for implanted neural prostheses. J. Biomed. Mater. Res. - Part B Appl. Biomater..

[32] Schmidt EM, McIntosh JS, Bak MJ (1988). Long-term implants of Parylene-C coated microelectrodes. Med. Biol. Eng. Comput..

[33] Takeuchi S, Ziegler D, Yoshida Y, Mabuchi K, Suzuki T (2005). Parylene flexible neural probes integrated with microfluidic channels. Lab Chip.

[34] Rodger DC (2008). Flexible parylene-based multielectrode array technology for high-density neural stimulation and recording. Sensors Actuators, B Chem..

[35] Hassler C, Boretius T, Stieglitz T (2011). Polymers for neural implants. Journal of Polymer Science, Part B: Polymer Physics.

[36] Song JS, Lee S, Jung SHS, Cha GC, Mun MS (2009). Improved biocompatibility of parylene‐C films prepared by chemical vapor deposition and the subsequent plasma treatment. J. Appl. Polym. Sci..

[37] Winslow BD, Christensen MB, Yang WK, Solzbacher F, Tresco PA (2010). A comparison of the tissue response to chronically implanted Parylene-C-coated and uncoated planar silicon microelectrode arrays in rat cortex. Biomaterials.

[38] Christensen MB, Wark HAC, Hutchinson DT (2015). A histological analysis of human median and ulnar nerves following implantation of Utah slanted electrode arrays. Biomaterials.

[39] de la Oliva, N., Navarro, X. & del Valle, J. Time course study of long-term biocompatibility and foreign body reaction to intraneural polyimide-based implants. *J*. *Biomed*. *Mater*. *Res*. *Part A* 1–29 (2017). 10.1002/jbm.a.36274 29052368

[40] Christensen MB (2014). The foreign body response to the Utah Slant Electrode Array in the cat sciatic nerve. Acta Biomater..

[41] Wurth S (2017). Long-term usability and bio-integration of polyimide-based intra-neural stimulating electrodes. Biomaterials.

[42] Vince V, Brelen ME, Delbeke J, Colin IM (2005). Anti-TNFa reduces the inflammatory reaction associated with cuff electrode implantation around the sciatic nerve. J. Neuroimmunol..

[43] Doloff JC (2017). Colony stimulating factor-1 receptor is a central component of the foreign body response to biomaterial implants in rodents and non-human primates. Nat. Mater..

[44] Luttikhuizen DT, Harmsen MC, Van Luyn MJA (2006). Cellular and molecular dynamics in the Foreign Body Reaction. Tissue Eng..

[45] Rodriguez A, Meyerson H, Anderson JM (2009). Quantitative *in vivo* cytokine analysis at synthetic biomaterial implant sites. J. Biomed. Mater. Res. - Part A.

[46] Baldwin L, Hunt JA (2008). The *in vivo* cytokine release profile following implantation. Cytokine.

[47] Sahin H, Wasmuth HE (2013). Chemokines in tissue fibrosis. Biochim. Biophys. Acta - Mol. Basis Dis..

[48] Myllymaa S (2010). Surface characterization and *in vitro* biocompatibility assessment of photosensitive polyimide films. Colloids Surfaces B Biointerfaces.

[49] Goda T, Konno T, Takai M, Ishihara K (2007). Photoinduced phospholipid polymer grafting on Parylene film: Advanced lubrication and antibiofouling properties. Colloids Surfaces B Biointerfaces.

[50] Jones JA (2007). Proteomic analysis and quantification of cytokines and chemokines from biomaterial surface-adherent macrophages and foreign body giant cells. J. Biomed. Mater. Res. - Part A.

[51] Ghosh I, Konar J, Bhowmick AK (1997). Surface properties of chemically modified polyimide films. J. Adhes. Sci. Technol..

[52] Grellmann, W. & Seidler, S. *Polymer Testing*. *Hanser Publishers*, *Munich* (Carl Hanser Verlag GmbH & Co. KG, 2013).

[53] del Valle, J., de la Oliva, N., Mueller, M., Stieglitz, T. & Navarro, X. Biocompatibility evaluation of parylene C and polyimide as substrates for peripheral nerve interfaces. In *2015 7th International IEEE/EMBS Conference on Neural Engineering (NER)* 442–445 (2015).

[54] Boretius T (2010). A transverse intrafascicular multichannel electrode (TIME) to interface with the peripheral nerve. Biosens. Bioelectron..

[55] Santos D, Wieringa P, Moroni L, Navarro X, Valle J (2017). Del. PEOT/PBT Guides Enhance Nerve Regeneration in Long Gap Defects. Adv. Healthc. Mater..

[56] de Medinaceli L, Freed WJ, Wyatt RJ (1982). An index of the functional condition of rat sciatic nerve based on measurements made from walking tracks. Exp. Neurol..

