# Supplementary material for: On the use of Parylene C polymer as substrate for peripheral nerve electrodes
Source: Sci Rep. 2018 Apr 13;8:5965. doi: 10.1038/s41598-018-24502-z (PMC5899141; doi:10.1038/s41598-018-24502-z)

# **On the use of Parylene C polymer as substrate for peripheral nerve electrodes**

Natàlia de la Oliva^1^, Matthias Mueller^2^, Thomas Stieglitz^2^, Xavier Navarro^1^, Jaume del Valle^1.3*^

^1^Institute of Neurosciences, Department of Cell Biology, Physiology and Immunology, Universitat Autònoma de Barcelona, and Centro de Investigación Biomédica en Red en Enfermedades Neurodegenerativas (CIBERNED), Bellaterra, Spain.

^2^Laboratory for Biomedical Microtechnology, Department of Microsystems Engineering IMTEK, Albert-Ludwig-University Freiburg, Freiburg, Germany

^3^Catalan Institute of Nanoscience and Nanotechnology (ICN2), CSIC and BIST, Campus UAB, Bellaterra, 08193 Barcelona, Spain

**Corresponding author**: Dr. Jaume del Valle, Unitat de Fisiologia Mèdica, Facultat de Medicina, Universitat Autònoma de Barcelona, E-08193 Bellaterra, Spain. E-mail: jaume.delvalle@uab.cat


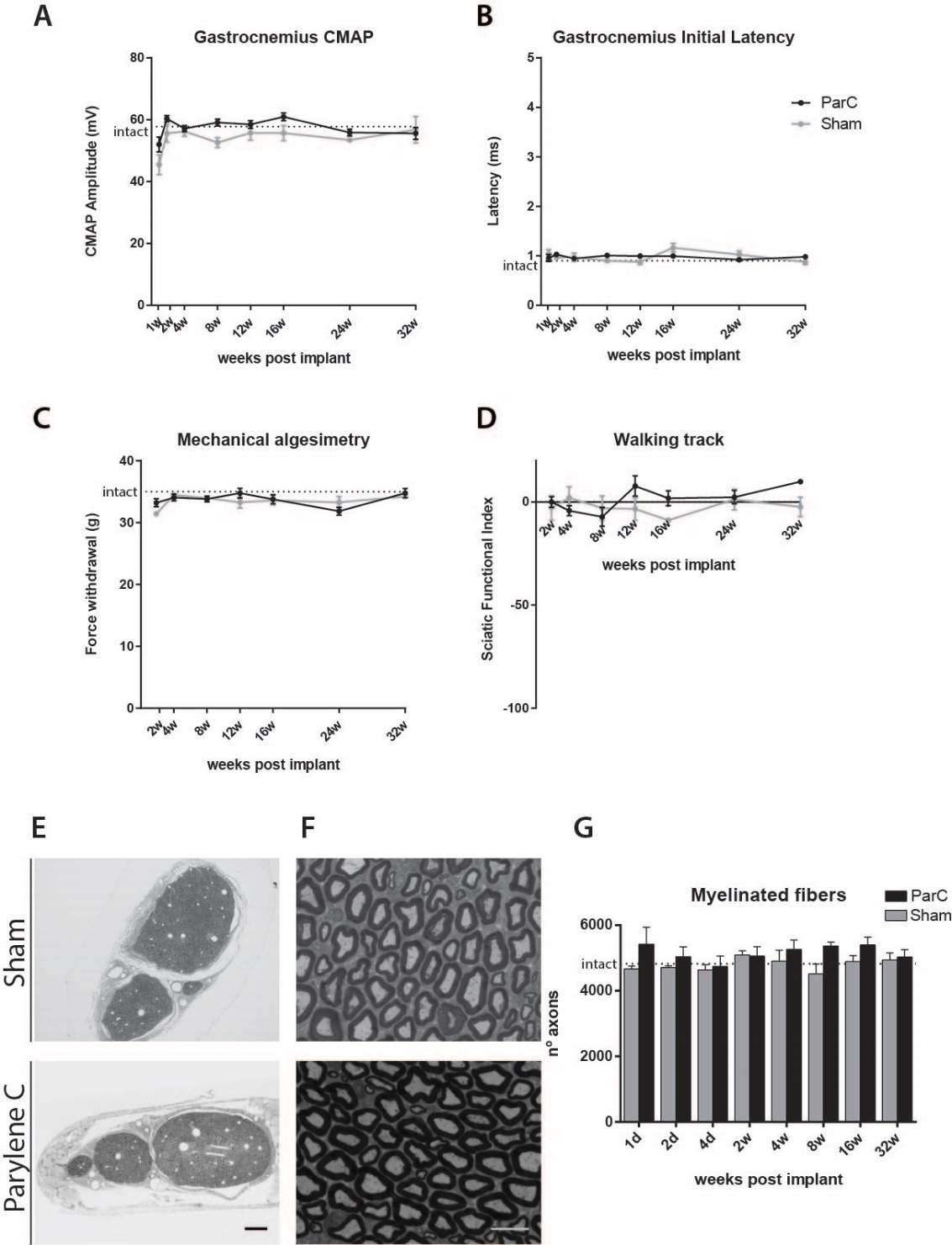


**Supplementary figure 1. Functional and histological evaluation** of implanted nerves. No differences were found in (A) amplitude or (B) latency of the gastrocnemius CMAP between Parylene C and sham groups. (C) No alterations in pain threshold assessed by Von Frey mechanical algesimetry in the central area of the paw due to the surgery or the device implanted. (D) No differences in SFI values of sham and Parylene C implanted animals. Dotted lines indicate values from intact animals. (E) Low and (F) high magnification of sham and implanted nerves. Scale bar in (E) = 150μm and (F) = 10μm. (G) No changes in the total number of myelinated fibers in the Parylene C implanted group in comparison to the sham group.

**Supplementary figure 2. FBGCs presence in the capsule around the Parylene C device in the nerve.** Representative images at (A) 2 and (B) 8 weeks post-implant. Two FBGCs are delineated. Scale bar = 20µm. Changes in (C) number and (D) diameter of FBGCs over time.


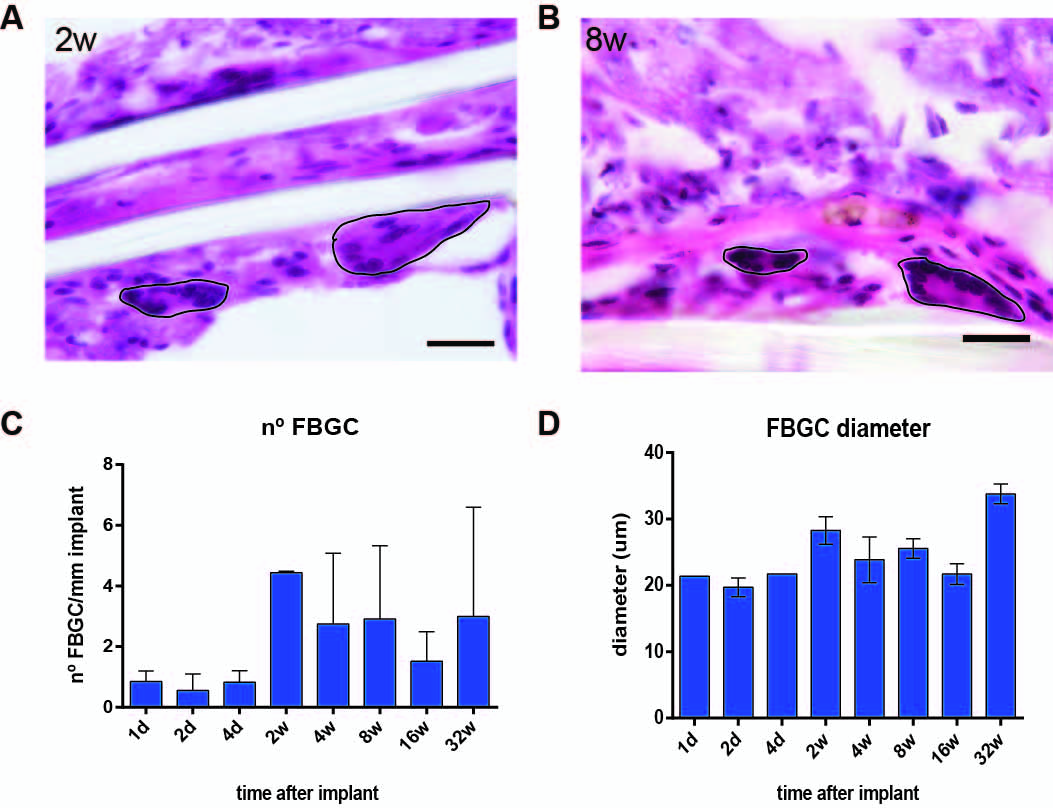


**Supplementary figure 3. Dynamic changes of cytokines levels** after sham or device implantation. Heatmaps (A-C) and ratio values (A’-C’) showing the changes in protein levels of relevant pro-inflammatory, anti-inflammatory and tissue remodeling-related factors in (A, A’) parylene, (B, B’) polyimide and (C, C’) sham implanted nerves. Results expressed as the mean of the ratio of each group and intact values. Crossed out squares mean no protein detected.


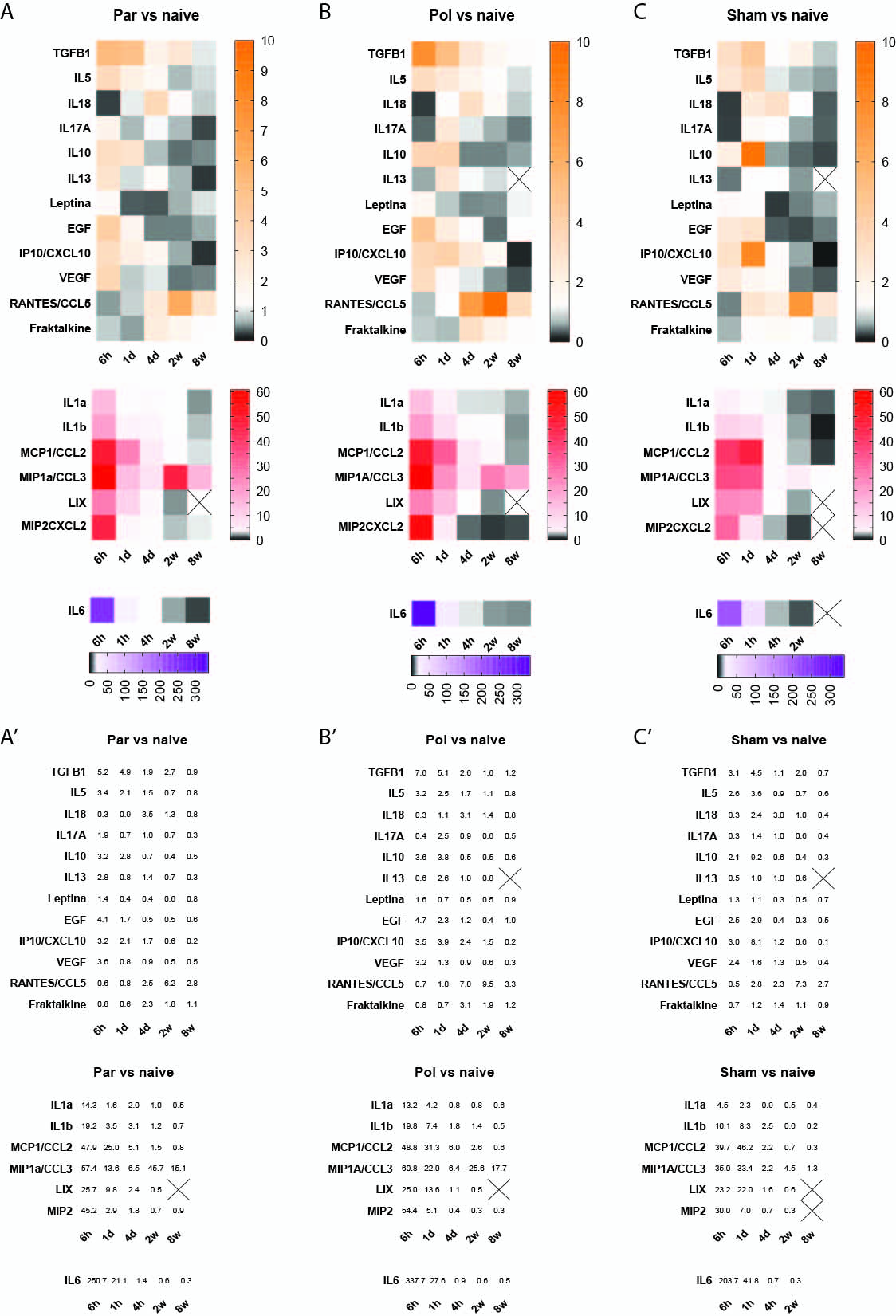


**Supplementary figure 4. Ratio values for cytokines levels** after device implantation. Ratio values of nerves with (A) Parylene C and (B) polyimide device implanted versus sham animals. (C) Ratio values of protein expression for Parylene C versus polyimide implanted nerves. Results expressed as the mean of the ratio between each group and sham or polyimide values. Crossed out squares mean no protein detected.


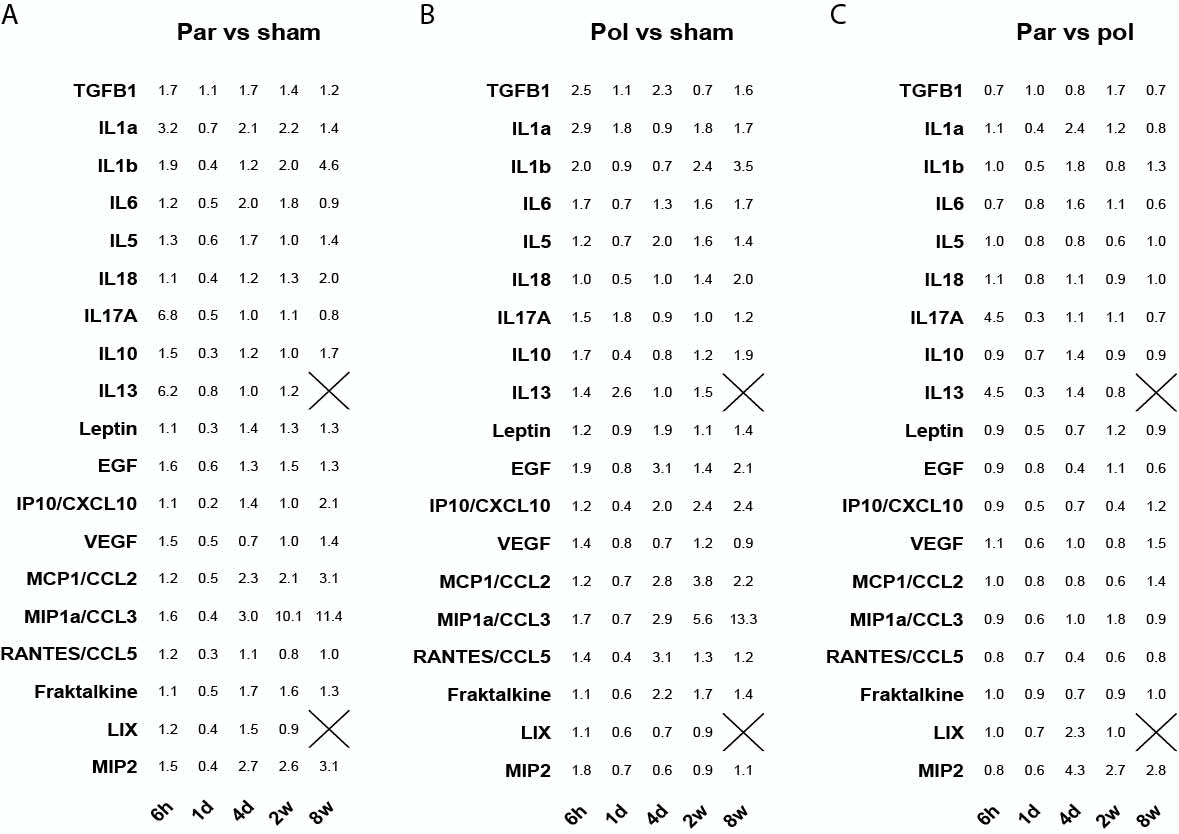

Supplement: Supplementary file 1 — Supplementary Figures [file 41598_2018_24502_MOESM1_ESM.docx]
